# Dual TMPRSS2:ERG Fusion in a Patient with Lung and Prostate Cancers

**DOI:** 10.3390/diagnostics10121109

**Published:** 2020-12-20

**Authors:** Francesca Giunchi, Francesco Massari, Annalisa Altimari, Elisa Gruppioni, Elisabetta Nobili, Michelangelo Fiorentino, Andrea Ardizzoni

**Affiliations:** 1 Department of Pathology, Azienda Ospedaliero-Universitaria di Bologna IRCCS Policlinico S.Orsola-Malpighi, Via Albertoni 15, 40138 Bologna, Italy; francesca.giunchi@aosp.bo.it (F.G.); annalisa.altimari@aosp.bo.it (A.A.); elisa.gruppioni@aosp.bo.it (E.G.); 2 Department of Clinical Oncology, Azienda Ospedaliero-Universitaria di Bologna IRCCS Policlinico S.Orsola-Malpighi, Via Albertoni 15, 40138 Bologna, Italy; fmassari79@gmail.com (F.M.); elisabetta.nobili@aosp.bo.it (E.N.); andrea.ardizzoni@aosp.bo.it (A.A.); 3 Department of Diagnostic Specialistic and Experimental Medicine, Alma Mater Studiorum University of Bologna School of Medicine, 40138 Bologna, Italy

**Keywords:** TMPRSS2:ERG fusion, prostate cancer, lung cancer, next-generation sequencing

## Abstract

The TMPRSS2:ERG fusion is considered prostate specific and has been rarely described in other tumors. We describe the case of a patient who developed lung and prostate cancers, both harboring the TMPRSS2:ERG fusion. The patient developed a cancer of the prostate with lymph node metastases and after two years a nodule of the thoracic wall. The histology and immunohistochemical profile of the two tumors were typical of prostate and lung cancers. The presence of the TMPRSS2:ERG fusion was demonstrated by next-generation sequencing on both malignancies, leading to the assumption that the lung nodule was a metastasis from the prostate cancer. The patient failed to respond to antiandrogen therapy, while chemotherapy for lung cancer led to a significant objective response. To our knowledge, this is the first case of a lung cancer harboring the TMPRSS2:ERG fusion, widening the spectrum of lung cancer-associated molecular alterations.

## 1. Introduction

The TMPRSS2:ERG fusion is the most frequent genetic alteration in prostate cancer (PCA) [1]. The gene encoding for the androgen-regulated transmembrane protease serine S2 (TMPRSS2) translocates with the E-twenty-six-related transcription factor ERG, leading to overexpression and oncogenicity of ERG [2]. The TMPRSS2:ERG fusion is considered prostate specific, and the pathogenesis of the gene fusion may be ascribed to higher levels of circulating androgens with potential direct effect on prostate cell DNA [3]. The TMPRSS2:ERG fusion can be investigated by fluorescence in situ hybridization or directly by sequencing the fusion RNA or indirectly by assessing the overexpression of the ERG protein by immunohistochemistry [4].

The occurrence of the TMPRSS2:ERG rearrangement in tumors other than the prostate is exceedingly rare, and therefore, the fusion can be considered pathognomonic for the diagnosis of PCA [5]. In the lung, the TMPRSS2:ERG fusion has been detected so far in a single case of small-cell carcinoma but never in non-small-cell lung cancer (NSCLC) [6]. Notwithstanding, the expression of TMPRSS2 in normal and cancer lung tissues has never been investigated, and the expression of ERG seems limited to endothelial normal cells [7].

Besides its diagnostic role, the presence of the TMPRSS2:ERG fusion has been variably and inconsistently associated with the outcome of PCA. While the prognostic value of the TMPRSS2:ERG fusion in PCA is debated, the role of TMPRSS2 as target of therapy has increased in the last weeks. The interest in the expression of the TMPRSS2 protein has recently risen since its recognition as the priming enzyme of the spike protein of the coronavirus SARS-CoV-2 for the entry into type II pneumocytes [8]. In fact, serine proteases such as TMPRSS2 act through the proteolytic cleavage of coronavirus spike proteins, allowing the entry of the virus into airway cells [9]. Camostat mesilate is a powerful inhibitor of serine proteases, including TMPRSS2, and it efficiently prevents infections with SARS-CoV-2 of human cells in vitro [7]. The effect of camostat mesilate on TMPRSS2 expressing cancer cells has never been investigated, but it represents a potential therapeutic target in human cancers whose main genetic alteration is the TMPRSS2:ERG fusion.

Here, we describe the first case, to our knowledge, of a patient who developed lung and prostate cancers, both harboring the same TMPRSS2:ERG fusion.

## 2. Materials and Methods

Surgical specimens were formalin fixed, paraffin embedded, and routinely processed for histological diagnosis. Three-micron-thick sections were cut from paraffin blocks and stained with hematoxylin and eosin and through immunohistochemistry. TTF-1, BerEP4, NKX3.1, and PSA antibodies were performed in all cases using an automated BenchMark ULTRA instrument (Roche-Ventana).

**Figure 1:**
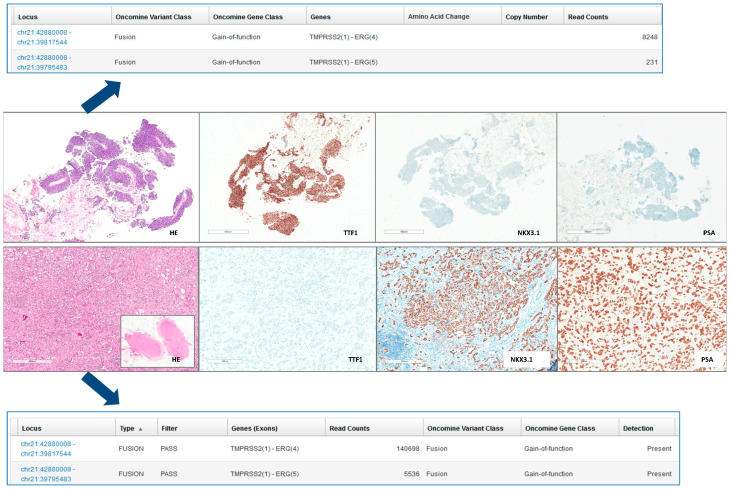
Histological, immunohistochemical, and NGS features of the lung (**top panel**) and prostate (**bottom panel**) lesions (100× magnification for all histological images). Blue arrows indicate the genetic profile corresponding to the histological appearance. HE: hematoxylin and eosin; TTF-1: thyroid transcription factor-1; PSA: prostate-specific antigen.

Next-generation sequencing (NGS) profiling was performed with the Oncomine 318 Focus Assay (Thermo Fisher Scientific, Waltham, MA, USA), which was carried out on an Ion GeneStudio S5 System (Thermo Fisher Scientific). The Focus Assay allows the detection of genetic variants across 52 cancer-relevant genes from tumor DNA and RNA, including single-nucleotide variations, copy number alterations, and gene fusions (Figure 1).

## 3. Results

A 77-year-old man, former smoker, was diagnosed with prostate adenocarcinoma in 2016 and treated with radiation therapy. In May 2017, following a PSA increase (5.9 ng/mL), the patient underwent radical prostatectomy and lymphadenectomy with the histological report of a stage pT3b N1 Mx R1 PCA and a single lymph node metastasis. The patient started adjuvant treatment with leuprorelin.

**Figure 2:**
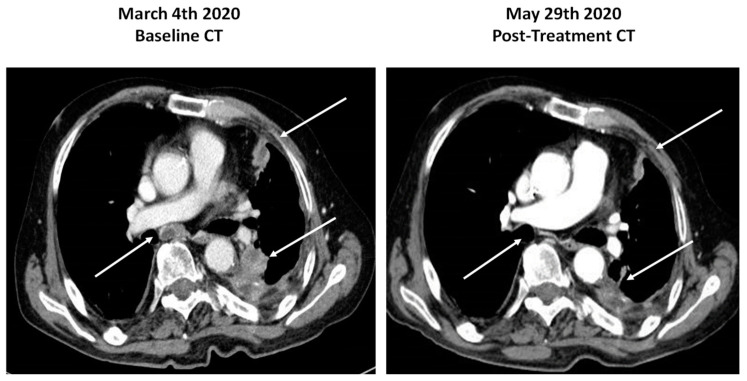
CT scans of the lung lesions before and after chemotherapy with carboplatin and pemetrexed. Arrows highlight the significant response to therapy of mediastinal, pleural, and lung lesions.

In May 2019, our patient was admitted to the emergency room for acute respiratory distress. A CT scan showed left pleural effusion, thickening, and nodules of the pleural surface, several mediastinal lymph node metastases, and a bone metastasis in the IX left rib (Figure 2 left). Pleural effusion cytology came back negative for malignancy. An ultrasound-guided bone biopsy of the lesion at the IX rib level was carried out. Histology revealed malignant cells with adenocarcinoma aggregation. The immunohistochemical workout was guided by the anamnesis of prostate cancer and included markers of prostatic differentiation (NKX3.1 and PSA) and markers of lung differentiation (TTF-1 and CK7). The cancer cells turned out to be highly immunoreactive for TTF-1 and BerEP4 and negative for the prostatic markers, leading to the diagnostic suspect of lung adenocarcinoma (Figure 1 top panel).

For routine molecular characterization of all newly diagnosed metastatic NSCLCs in our hospital, a next-generation sequencing (NGS) profiling was carried out and revealed the TMPRSS2:ERG as a unique alteration (covered by the panel) of the rib lesion (Figure 1 top panel).

For thorough diagnostic accuracy, the NGS panel and diagnostic immunohistochemistry were also applied to the prostate cancer lymph node metastasis from the radical prostatectomy of 2017. The prostate cancer tissue was positive for the prostate immunohistochemical markers (NKX3.1, PSA) and negative for the lung markers (TTF-1, BerEP4) but harbored the TMPRSS2:ERG fusion as the only molecular alteration (Figure 1 bottom panel).

In August 2019, given the increase of serum PSA (2.3 ng/mL), the patient underwent multiple pleural biopsies to exclude the concomitant presence of lung cancer and prostate cancer metastases. The histological diagnosis revealed again a poorly differentiated adenocarcinoma positive for the lung markers TTF-1 and CK7 (see Figure S1) and negative for the prostatic markers (PSA and NKX3.1). The differential diagnosis suggested either a primitive pulmonary cancer or a metastatic localization of neuroendocrine castration-resistant prostate adenocarcinoma. Notwithstanding, given the high specificity of the TMPRSS2:ERG fusion for prostate cancer and the clinical data (progressive PSA increase after radical prostatectomy and during hormone therapy) and the absence of typical parenchymal lung lesions, the final decision oriented for a pleural metastasis from prostate adenocarcinoma with a neuroendocrine component. Therefore, a first-line therapy with enzalutamide was started.

In January 2020, due to recurrent pleural effusion and respiratory failure, the patient was admitted to the thoracic surgery ward and a talc pleurodesis was performed. The CT scan showed a progressive disease with several pleural metastases and bone metastases of the IX and V left ribs with muscular infiltration, despite a PSA level of 1 ng/mL.

In February 2020, the patient underwent another CT-guided pleural biopsy with a histological diagnosis of a papillary poorly differentiated adenocarcinoma (still immunoreactive for TTF-1 and negative for NKX3.1, p40, chromogranin, and calretinin). Histology always excluded pleural mesothelioma. The histology report together with the lack of response to enzalutamide favored the diagnosis of peripheral NSCLC with pleural and rib infiltration, harboring the same TMPRSS2:ERG rearrangement found in the prostate cancer. The patient was therefore submitted to first-line chemotherapy with carboplatin and pemetrexed. In June 2020, after six cycles of chemotherapy, the clinical condition of the patient improved, and a control CT scan showed significant objective response of the pleural lesions (Figure 2 right).

## 4. Discussion

We describe what is, to our knowledge, the first case of NSCLC harboring the TMPRSS2:ERG fusion. Our index patient experienced a troublesome diagnostic and clinical workflow mainly due to the concomitant occurrence of prostate and lung cancers and the peculiar clinical presentation of the lung tumor. This case report leads to the following considerations.

First, the TMPRSS2:ERG fusion is not pathognomonic of PCA. The diagnostic sensitivity of the fusion for prostate cancer is actually low since it is detectable in approximately 50%–60% of tumors. However, the specificity of the TMPRSS2:ERG fusion for PCA is generally considered very high, and its detection in challenging cases is a guide to address diagnosis and therapy towards a prostate malignancy whatsoever. The same happened to our patient, who was treated initially for PCA even if the histology of the pleural lesions was more suggestive of a primary lung cancer. The recent history of a PCA harboring the same TMPRSS2:ERG fusion in lymph node metastases and the absence of a typical lung primary lesion led the oncologists to pursue with the therapy for metastatic PCA. The lack of radiological objective response to androgen blockade and enzalutamide, despite PSA reduction, together with the results of repeated tissue biopsies all pointing to a histological diagnosis of primary adenocarcinoma of the lung, finally led us to treat the patient with NSCLC-oriented chemotherapy. The objective response to the lung-oriented chemotherapy confirmed the diagnosis of NSCLC ex juvantibus.

Second, the patient would have been probably treated for lung cancer from the onset of the thoracic lesion if the TMPRSS2:ERG fusion had not been found using the multi-gene NGS panel, and a single specific gene molecular test approach would have been applied instead. In the current era of widespread molecular cancer profiling with NGS panels, it should be considered that the occurrence of rare or unexpected genetic variants may be sometimes misleading. The advantages of large molecular cancer profiling are clearly greater than its drawbacks, but data coming from wide genetic analyses must be always considered carefully. Multidisciplinary molecular tumor boards for the cross discussion of histological, molecular, imaging, and clinical findings are highly required to avoid misinterpretations.

Finally, the TMPRSS2:ERG fusion has to be considered a driver genetic alteration also associated with NSCLC. The genetic profiles of prostate and lung cancers are clearly distinct in most cases with the TMPRSS2-ERG fusion being by far the most common genetic alteration in prostate cancer [10]. By contrast, the most common genetic alterations in NSCLC are either smoke related (RAS family) or p53 related [11]. Although rare, our observation could be replicated by others, particularly with the rapid shift of lung cancer molecular profiling towards NGS panels almost everywhere. The fusion has recently been found in a case of SCLC [6]. Recent advances on the key role of TMPRSS2 in the entry of SARS-CoV-2 in alveolar cells confirms the presence of *TMPRSS2* expression in lung tissues. This finding also bears important therapeutic implications since TMPRSS2 inhibitors such as camostat mesilate are also potentially active in tumors harboring the TMPRSS2:ERG fusion, regardless of the tumor origin.

Whether TMPRSS2 acts under the androgen regulation also in the lung and the pathogenesis of the TMPRSS2:ERG fusion in tissues other than the prostate must be elucidated in further biological studies. In the meantime, we recommend considering the occurrence of this novel actionable gene fusion in non-prostate-related malignancies.

## Supplementary Materials

The following are available online at https://www.mdpi.com/2075-4418/10/12/1109/s1: Figure S1: immunohistochemical expression of CK7 and TMPRSS2.
