# Supplementary material for: Dual TMPRSS2:ERG Fusion in a Patient with Lung and Prostate Cancers
Source: Diagnostics (Basel). 2020 Dec 20;10(12):1109. doi: 10.3390/diagnostics10121109 (PMC7765862; doi:10.3390/diagnostics10121109)
Supplement: Supplementary file 1 [file diagnostics-10-01109-s001.pdf]

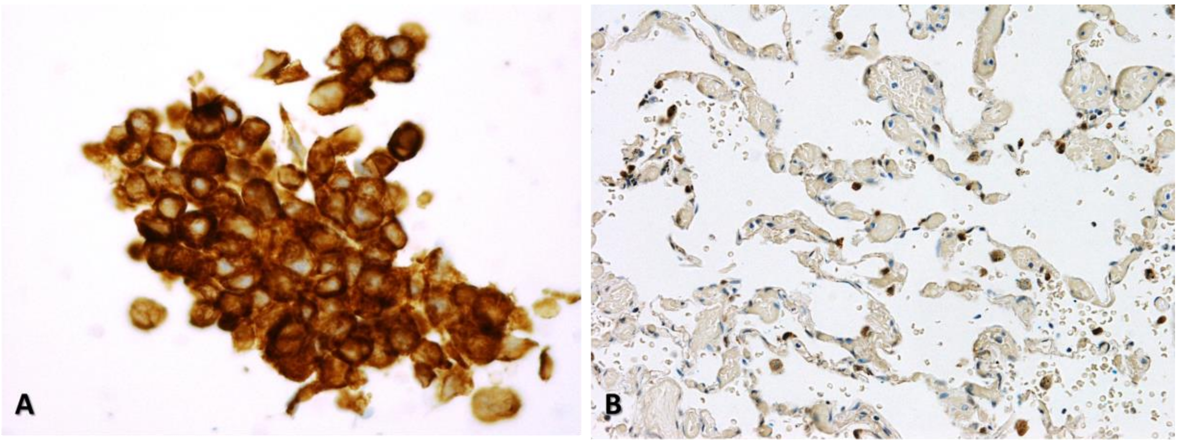

Supplemetary Figure 1.

(**A**) Positive immunostaining for CK7 of the second diagnostic biopsy of the lung mass (40x magnification); (**B**) Immunohistochemistry for TMPRSS2 in a normal lung specimen. The immunostaining is positive in scattered type II pneumocytes (20x magnification).
